# Intercomparison of Soil Moisture Retrieved from GNSS-R and from Passive L-Band Radiometry at the Valencia Anchor Station

**DOI:** 10.3390/s19081900

**Published:** 2019-04-22

**Authors:** Cong Yin, Ernesto Lopez-Baeza, Manuel Martin-Neira, Roberto Fernandez-Moran, Lei Yang, Enrique A. Navarro-Camba, Alejandro Egido, Antonio Mollfulleda, Weiqiang Li, Yunchang Cao, Bin Zhu, Dongkai Yang

**Affiliations:** 1 Department of Atmospheric Physics, Nanjing University of Information Science and Technology, Nanjing 210044, China; binzhu@nuist.edu.cn; 2 Meteorological Observation Centre, China Meteorological Administration, Beijing 100081, China; caoyc@126.com; 3 Faculty of Physics, Earth Physics and Thermodynamics Department, Climatology from Satellites Group, University of Valencia, Burjassot, 46100 Valencia, Spain; Ernesto.Lopez@uv.es (E.L.-B.); roberto.fernandez@uv.es (R.F.-M.); 4 European Space Agency, ESA-ESTEC, 2200 AG Noordwijk, The Netherlands; Manuel.Martin-Neira@esa.int; 5 College of Information Science and Engineering, Shandong Agricultural University, Tai’an 271018, China; yanglei_sdau@163.com; 6 IRTIC, University of Valencia, C/. Catedrático José Beltrán, 2, 46980 Paterna, Spain; Enrique.Navarro@uv.es; 7 Starlab-Living Science, 08035 Barcelona, Spain; alejandro.egido@noaa.gov (A.E.); antonio.mollfulleda@neuroelectrics.com (A.M.); 8 Earth Observation Research Group, Institute of Space Sciences (ICE, CSIC), 08193 Barcelona, Spain; weiqiang.li.buaa@gmail.com; 9 School of Electronic and Information Engineering, Beihang University, Beijing 100083, China; yangdongkai@sina.com

**Keywords:** ELBARA-II radiometer, GNSS-R, L-band radiometry, Oceanpal, soil moisture, Valencia Anchor Station

## Abstract

In this paper, the SOMOSTA (Soil Moisture Monitoring Station) experiment on the intercomparison of soil moisture monitoring from Global Navigation Satellite System Reflectometry (GNSS-R) signals and passive L-band microwave radiometer observations at the Valencia Anchor Station is introduced. The GNSS-R instrument has an up-looking antenna for receiving direct signals from satellites, and a dual-pol down-looking antenna for receiving LHCP (left-hand circular polarization) and RHCP (right-hand circular polarization) reflected signals from the soil surface. Data were collected from the three different antennas through the two channels of Oceanpal GNSS-R receiver and, in addition, calibration was performed to reduce the impact from the differing channels. Reflectivity was thus measured, and soil moisture could be retrieved. The ESA (European Space Agency)-funded ELBARA-II (ESA L Band Radiometer II) is an L-band radiometer with two channels with 11 MHz bandwidth and respective center frequencies of 1407.5 MHz and 1419.5 MHz. The ELBARAII antenna is a large dual-mode Picket horn that is 1.4 m wide, with a length of 2.7 m with −3 dB full beam width of 12° (±6° around the antenna main direction) and a gain of 23.5 dB. By comparing GNSS-R and ELBARA-II radiometer data, a high correlation was found between the LHCP reflectivity measured by GNSS-R and the horizontal/vertical reflectivity from the radiometer (with correlation coefficients ranging from 0.83 to 0.91). Neural net fitting was used for GNSS-R soil moisture inversion, and the RMSE (Root Mean Square Error) was 0.014 m^3^/m^3^. The determination coefficient between the retrieved soil moisture and in situ measurements was R^2^ = 0.90 for Oceanpal and R^2^ = 0.65 for Elbara II, and the ubRMSE (Unbiased RMSE) were 0.0128 and 0.0734 respectively. The soil moisture retrievals by both L-band remote sensing methods show good agreement with each other, and their mutual correspondence with in-situ measurements and with rainfall was also good.

## 1. Introduction

L-band microwaves have very good advantages in soil moisture remote sensing, for being unaffected by the atmosphere (clouds, aerosols, etc.), and for the ability to penetrate vegetation, except in very dense forests. Using GNSS (Global Navigation Satellite System)-reflected signals for remote sensing applications was put forward by ESA in 1993 [1]. Extending GNSS-R applications toward soil moisture remote sensing was first proposed by Zavorotny and Voronovich [2] in 2000. To measure GNSS signals reflected from the land surface, the well-known flight campaign SME02 was launched by NASA and the University of Colorado in 2002 [3]. LEiMON (Land Monitoring with Navigation Signals) experimental field campaign was carried out by ESA and Starlab in 2009 to investigate the interaction between land surface parameters such as soil moisture, surface roughness, and vegetation biomass, and the scattered GNSS signal characteristics [4]. A brand-new method was proposed by Larson [5,6], who verified that multipath signals could be used to infer near-surface soil moisture. A single up-looking antenna was used to receive the multipath combination of direct signal and reflected signals, the latter varying with soil moisture. A physical model of GNSS direct and reflected signal interference was built by Zavorotny and Larson [7]. Rodriguez-Alvarez et al. [8] presented the measurements applying the interference pattern technique (IPT) for soil moisture and vegetation height retrievals over vegetation-covered soils. The potential of sensing land surface properties with spaceborne GNSS receivers have been demonstrated with the TDS-1 (TechDemoSat-1) and CYGNSS (Cyclone Global Navigation Satellite System) observations [9,10,11]. 

L-band radiometry is one of the most efficient techniques to monitor surface soil moisture on a global scale. Thus, the European space agency launched the Soil Moisture and Ocean Salinity (SMOS) mission in 2009 with a passive L-band radiometer dedicated to making global observations of soil moisture over land as well as salinity over oceans [12], and the NASA Soil Moisture Active and Passive (SMAP) satellite mission was launched in 2015 to provide global mapping of soil moisture using an L-band (active) radar and an L-band (passive) radiometer [13]. Both missions have been successful in providing surface soil moisture on a global scale from a space platform. For the ground referencing, the ELBARA-II radiometer was specifically designed for the validation of SMOS data and products and for the further improvement of the radiative transfer models used in the soil moisture retrieval algorithms [14]. Several ground experiments were carried out with an ELBARA-II radiometer in different regions [15,16,17,18,19]. Further steps were given by Chew and Small [9] who quantified the relationship between forward scattered GNSS signals recorded by the CYGNSS constellation and the SMAP soil moisture. Alonso-Arroyo et al. [20] also compared microwave radiometry and GNSS-R observations conducted for different soil moisture conditions from an airborne experiment.

In this work, the SOMOSTA (Soil Moisture Monitoring Station) long-term experiment at the Valencia Anchor Station (2014–2016) is introduced. This experiment is fruitfully framed within the ESA-China joint program of collaboration on GNSS-R. Based on the long-term measurements with Oceanpal GNSS reflectometry and ELBARA-II passive L-band radiometer, the correlation between the two sets of measurements was analyzed.

## 2. Materials and Methods

### 2.1. Field Campaign at the Valencia Anchor Station

The Valencia Anchor Station validation site is located on the Utiel-Requena plateau, 80 km west of the city of Valencia, in Spain, and the SOMOSTA experiment took place at the Valencia Anchor Station MELBEX (Mediterranean L-Band characterization Experiment) site, within the El Renegado vineyard area with coordinates (39°31′17.98″ N, 1°17′29.29″ W) and an altitude of 800 m. During this experimental campaign, the ESA GNSS-R Oceanpal [21] antenna was installed on the same tower as the ESA ELBARA-II passive microwave radiometer, the Ocenapal 11 m high from the ground, and both measuring instruments having a similar field of view. The Oceanpal Radio Frequency Unit (ORFU) and Data Management Unit (DMU) was installed at a small protected hut at the bottom of the tower.

**Figure 1:**
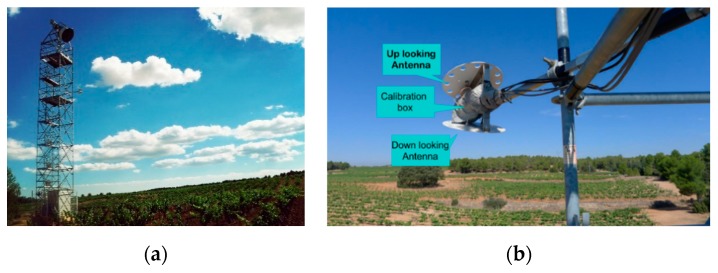
(**a**) ELBARA-II L-band radiometer tower also holding the Oceanpal GNSS-R antennas over a vineyard field at El Renegado MELBEX site at the Valencia Anchor Station; (**b**) Detail of the GNSS-R deployment indicating the essential elements.

Figure 1 shows the experimental set-up composed of the ELBARA-II L-band radiometer tower, also holding the Oceanpal GNSS-R antennas that are shown in detail in Figure 1b.

**Figure 2:**
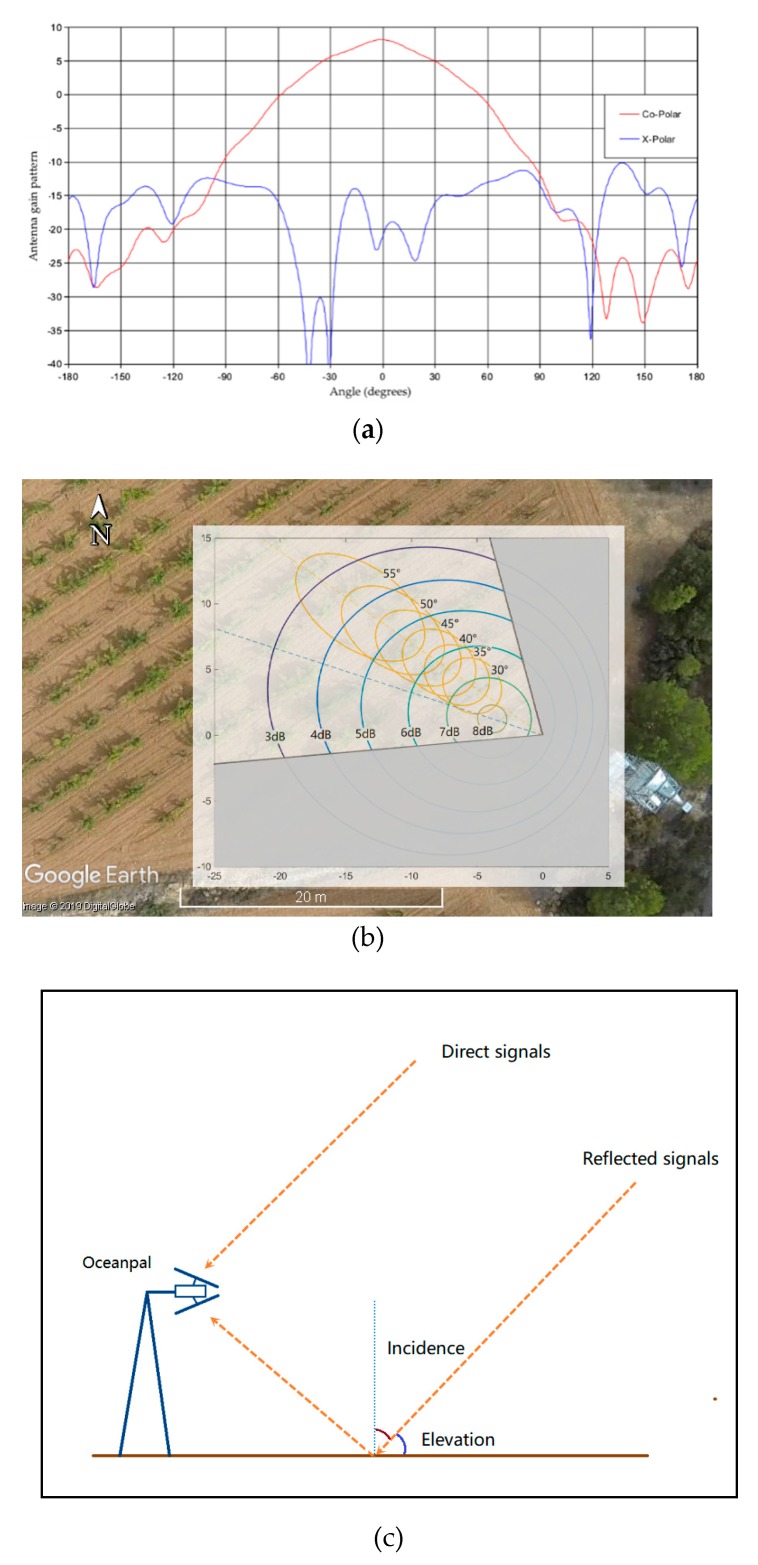
(**a**) Oceanpal antenna gain pattern; (**b**) projection of Oceanpal gain pattern and ELBARA-II footprint overlay on Google Earth. The upper layer shows the projection of the Oceanpal down-looking antenna’s gain over the soil surface, together with the ELBARA footprint, and the bottom layer is a photo taken by a camera drone overlay on the Google Earth base map; (**c**) The incidence angle is the complementary angle of elevation, as shown in the graph.

According to Figure 1b, the GNSS-R instrument has an up-looking antenna for receiving direct signals in RHCP (Right-Hand Circular Polarization) from GNSS satellites, and a down-looking antenna for receiving LHCP (Left-Hand Circular Polarization) and RHCP-reflected signals from the soil surface. The boresight direction of both antennas was pointing 20° away from the tower to reduce tower impact and for a larger observation range. The direction of the Oceanpal antenna is about 285° towards northwest (360° is the north). All three antennas have the same gain pattern, as shown in Figure 2a. Figure 2b combines the observation geometry from both instruments and the GNSS-R antenna pattern overlay on Google Earth. The yellow ellipses correspond to ELBARA-II footprints for incidence angles 30°, 35°, 40°, 45°, 50°, 55°, the azimuth was fixed to 305°, and the blue ellipses correspond to projections of Oceanpal gain patterns. The upper layer shows the projection of the Oceanpal down-looking antenna’s gain over the soil surface, together with the ELBARA-II footprint, the bottom layer is a photo taken by a camera drone overlaid on the Google Earth base map. The visible area of Oceanpal is within 20 m from the tower, and the azimuth range for observation is 265° to 345°.

**Figure 3:**
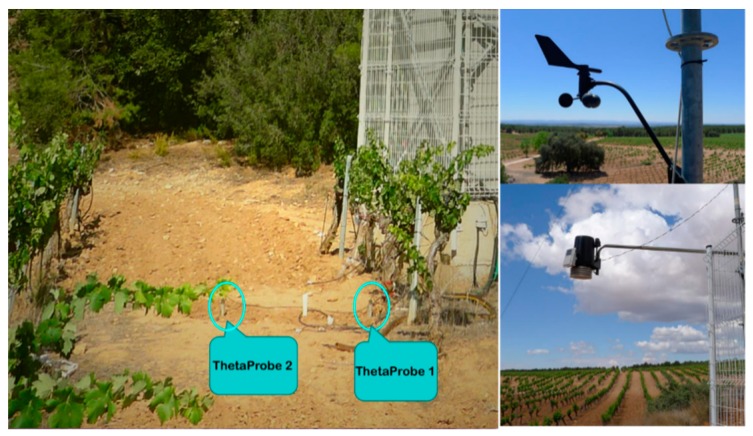
Soil moisture ThetaProbe sensors and Davis Vantage Pro2 Plus meteorological station.

Two soil moisture ThetaProbe sensors [22], type ML2x from the company Delta-T Devices, UK, were placed at the bottom of the tower. As displayed in Figure 3, the ThetaProbe 1 is close to a vine stump, and the ThetaProbe 2 is in the middle of two rows, both of them measuring soil moisture for top 5 cm. The averages of the measurements performed with these two probes were assumed to be representative of the soil moisture conditions in the MELBEX-III field site, and thus considered as the soil moisture reference in this study. A Davis Vantage Pro2 Plus weather station [23] was installed on the tower, measuring rainfall, temperature, humidity, and solar radiation.

### 2.2. Measurements and Data Processing

Unlike the ELBARA-II radiometer that measures brightness temperature to calculate soil reflectivity and permittivity, the GNSS reflectometry instrument measures direct signals from the GNSS satellites and their reflected signals from the soil surface, thus directly providing surface soil reflectivity. According to the Oceanpal instrument design, the reflected LHCP signals were acquired in channel 1, and direct signals in RHCP were acquired in channel 2, in the first measurement stage (1-LD). In the second stage (2-DR), direct signals were acquired in channel 1, and the reflected RHCP signals were acquired in channel 2, as shown in Table 1. Thus, the calibration box has two functions. Firstly, we could collect signals from 3 different antennas with two input channels; secondly, a calibration process was performed to reduce the impact from the two different channels [24]. Since the LHCP signals were considered to be more sensitive to soil moisture variation, a longer measuring time was set for the first measurement mode.

LHCP and RHCP reflectivities could then be calculated from equations

*K = mPD2/mPD*1 (1)

*Reflectivity_RL = (mPR1/mPD1)/K* (2)

*Reflectivity_RR = (mPR2/mPD2) × K* (3)

where *K* is the ratio of direct waveform peak power from the two different measurement stages, *mPD1* and *mPD2* are the direct waveform mean peak power measured in the two different measuring modes, and *mPR1* and *mPR2* represent the LHCP/RHCP-reflected waveform mean peak power measured in the different measuring modes. For GNSS measurements, the amplitude could be associated with soil properties.

**Figure 4:**
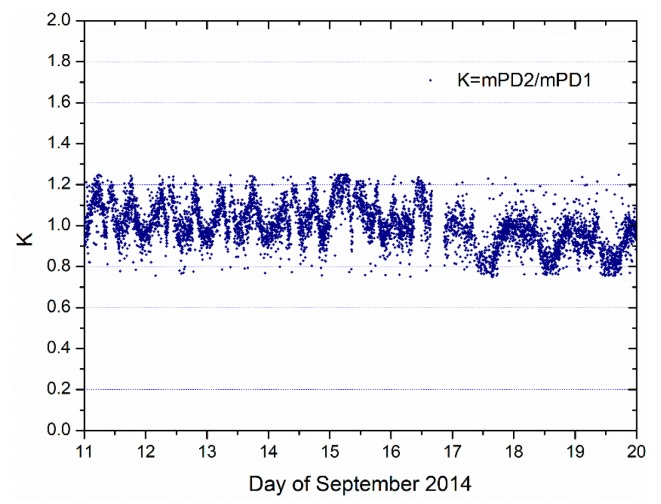
Calibration parameter *K* plot over time.

The direct signal waveform peak power ratio between consecutive measurements stages is shown in Figure 4. This represents the estimated calibration constant used for the relative calibration of the two instrument’s channels. For each data take, the calibration constant is computed for each PRN (pseudo-random noise) between two consecutive measurement stages [24]. The power ratio among channels was concentrated around 1, with an average value of 1.03 and a mean absolute deviation of 0.091 over the whole observation period (August 2014–May 2016). The fluctuations along time, could be caused by changes in the channel gain due to temperature variations and other environmental factors [24].

### 2.3. Soil Moisture Retrieval Algorithms for GNSS-R and ELBARA-II

Neural net fitting [25] was used for GNSS-R soil moisture inversion. The chosen neural network architecture consisted of one input layer, one hidden layer, and the output layer. The transfer function used was tansig and the number of nodes was 8. In total, 238 samples of GNSS daily measurements were used as input, and 238 samples of soil moisture measurements by in-situ probes were used as target. Randomly, 70% samples were selected for training, 15% were used to measure network generalization and to halt training when generalization stopped improving. The last 15% samples had no effect on training and were used for validation by providing an independent measure of the network performance during and after training.

**Figure 5:**
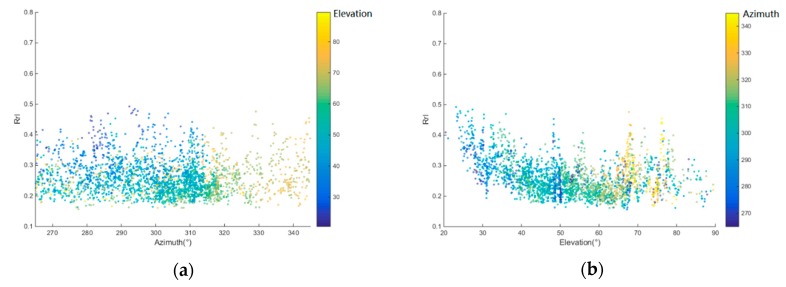
LHCP reflectivity vs azimuth angle (**a**) and vs elevation angle (**b**).

Besides soil moisture, vegetation and soil roughness also have an impact at L-band. In this paper, soil roughness was considered to be constant, and vegetation was assumed to have a homogeneous distribution. 250 m resolution MODIS (Moderate Resolution Imaging Spectroradiometer) EVI (Enhanced Vegetation Index) data were used for vegetation estimation. Spline interpolation was used to obtain daily EVI. Figure 5a, b shows LHCP reflectivity variation with azimuth and elevation. The data was measured in August 2014, with high vegetation, without any precipitation, and the soil moisture was constant at 0.10~0.11 m^3^/m^3^. The dependence of Rrl(LHCP reflectivity) with elevation is more obvious. So, we considered the azimuthal effect negligible. It was also assumed that the soil moisture diurnal variation was not obvious, so we could take the average over the Oceanpal measured LHCP/RHCP reflectivity with same incidence in one day.

ELBARA-II is a dual polarization L-band microwave radiometer which measures TB (brightness temperature) at two channels (1400–1418 MHz and 1409–1427 MHz) [14]. The observed brightness temperature at H or V polarization TB*p* (*p* = H, V) used in this study are averages of the brightness temperature values measured in both channels, which are part of the protected part of the microwave L-band. The radiometer was placed on a 15 m high platform over the vineyard to measure TB_V_ and TB_H_ automatically for different observation angles every 30 min. For the soil moisture retrieval, we used measurements at angles = 30°, 35°, 40°, 45°, 50°, and 55°, relative to nadir [26]. 

ELBARA-II soil moisture retrievals were based on the L-MEB model [26],

(4) $$TB_{p}\left(\theta \right)=T_{GC}\left[1-r_{Gp}^{\prime }\left(\theta \right)exp\left(-2\tau_{NAD}\frac{\cos^{2}\left(\theta \right)+tt_{p}\sin^{2}\left(\theta \right)}{\cos\left(\theta \right)}-H_{R}\cos^{N_{Rp}}\left(\theta \right)\right)\right]$$

where $\tau_{NAD}$ is the optical thickness, $tt_{V}$ and $tt_{H}$ are parameters used to quantify the dependence of $\tau_{p}$ on the incidence angle, and $N_{Rp}$ governs the changes in the angular dependence of reflectivity. For the specific case when $N_{RV}=N_{RH}=-1$, and vegetation is assumed as isotropic $tt_{H}=tt_{V}=1$, the Equation (4) was simplified as:

(5) $$TB_{p}\left(\theta \right)=T_{GC}\left[1-r_{Gp}^{\prime }\left(\theta \right)exp\left(-2TR/\cos\left(\theta \right)\right)\right]$$

where

(6) $$TR=\tau_{NAD}+H_{R}/2$$

In that case, vegetation (through the $\tau_{NAD}$ parameter) and roughness effects (through the HR parameter) can be combined in the single parameter TR, defined in Equations (5) and (6). $r_{Gp}^{\prime }\left(\theta \right)$ is reflectivity of a plane (specular) surface, which was computed from the Fresnel equations as a function of *θ* and of the soil dielectric constant ε, which in turn was computed as a function of soil moisture, soil effective temperature (T_GC_), and soil texture in terms of clay fraction [27]. For the MELBEX-III site, Juglea et al. [28] estimated the following soil fractions: sand (45%), silt (29%), and clay (26%). The values of the soil effective temperature were obtained from the ERA-INTERIM 0–7 cm soil temperature product from ECMWF (European center for medium range weather forecasting). This product has a spatial resolution of 1.5 degrees and a temporal resolution of three hours. The inversion is based on the minimization of a cost-function (CF) using a least-squares iterative algorithm [26].

## 3. Results

### 3.1. Waveform from Different GPS Satellites

**Figure 6:**
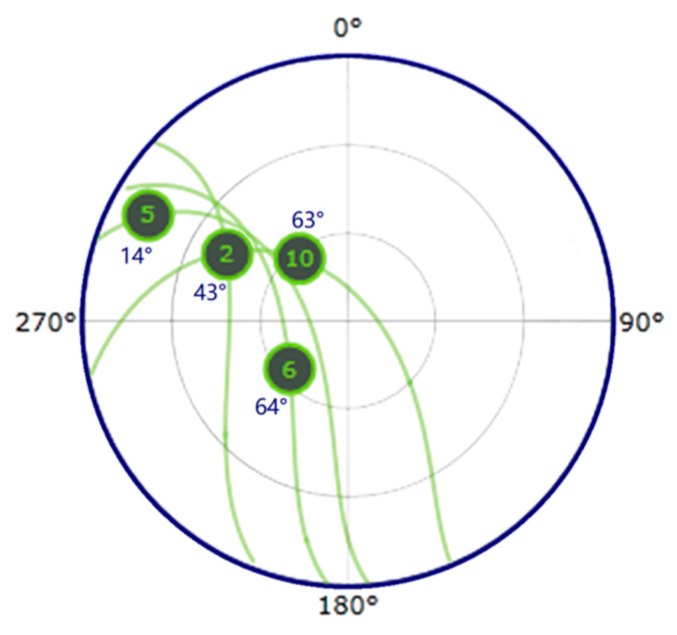
Skyplot at 22:00 UTC on 9th September, 2014 showing the in-view GPS satellites available and the respective elevation angles.

With the purpose of understanding how the reflected signal varies with the change of soil moisture and GNSS satellite elevation, some raw data from the GPS in-view satellites PRN 02, PRN05, PRN06 and PRN 10 (Figure 6) were selected for the days 8 and 9, September 2014. Soil moisture was constant at 0.1 m^3^/m^3^ until 21:00 UTC on 9th September, when 11 mm precipitation occurred and soil moisture raised to 0.22 m^3^/m^3^. Figure 6 then shows the GPS satellites skyplot of the MELBEX ELBARA-II site at 22:00 UTC on 9th September 2014.

**Figure 7:**
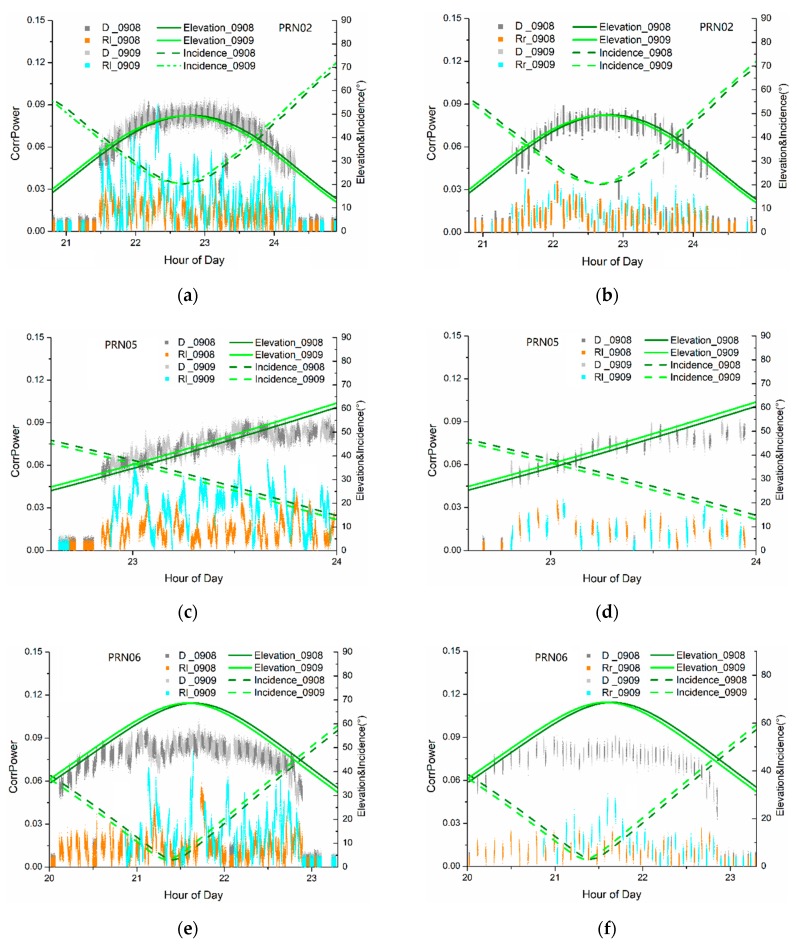

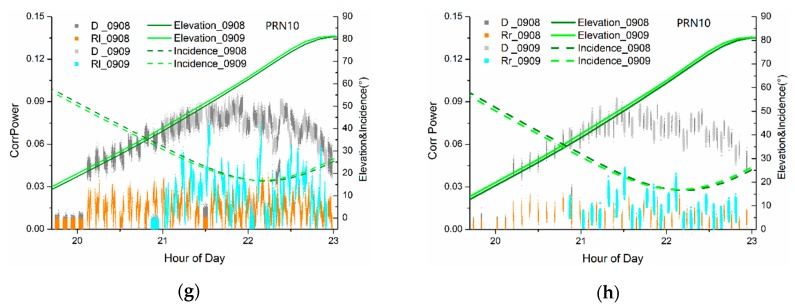
Correlation power of the direct and reflected signals in contrast with the elevation and incidence angles with respect to the antenna for different PRNs. (**a**) Direct, D, and LHCP reflected signals, Rl, of PRN 02, (**b**) direct, D, and RHCP reflected signals, Rr, of PRN 02, (**c**) direct, D, and LHCP reflected signals, Rl of PRN 05, (**d**) direct, D, and RHCP reflected signals, Rr of PRN 05, (**e**) direct, D, and LHCP reflected signals, Rl of PRN 06, (**f**) direct, D, and RHCP reflected signals, Rr of PRN 06, (**g**) direct, D, and LHCP reflected signals, Rl of PRN 10, (**h**) direct, D, and RHCP reflected signals, Rr of PRN 10. The 0908 and 0909 respectively represent 8th and 9th September 2014 (As an exception, in this figure, the incidence angle represents the angle between antenna bore sight and the reflected signal).

Figure 7 shows the correlation power of direct and LHCP/RHCP-reflected signals of four GPS satellites on two different days with different soil moisture. It also includes the satellite elevations and incidence angles to antenna (the angle between the incident signal and the antenna boresight). According to Figure 7, the correlation power of direct signals (D) were considered to be very stable because they only depend on the antenna gain pattern. In contrast, the correlation power of LHCP-reflected signals (Rl) were significantly affected by soil moisture. In Figure 7b,d,f,h, the correlation power of RHCP-reflected signals (Rr) also increase with rising soil moisture, but less obviously. It is worth noting that the signals with incidence angle larger than 45°~50°, for both direct and reflected signals have very low correlation power, lower than 0.015. This might be caused by antenna multipath reduction and, therefore, all direct signal data with correlation power lower than 0.015 were discarded during data processing.

### 3.2. Correlation Between Oceanpal GNSS and ELBARA-II Radiometer Measurements

The measurements lasted from August 2014 to May 2016, and there were some gaps due to observation interruptions of different kinds. During the measurement period, there were 238 days which had all GNSS LHCP reflectivity (Rrl), RHCP reflectivity (Rrr), ELBARA-II, and in-situ measurements.

**Figure 8:**
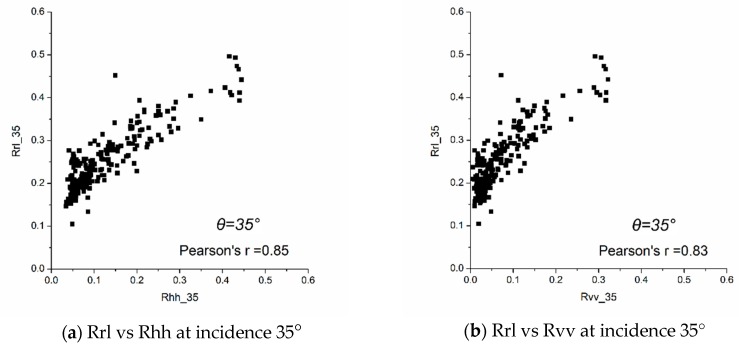

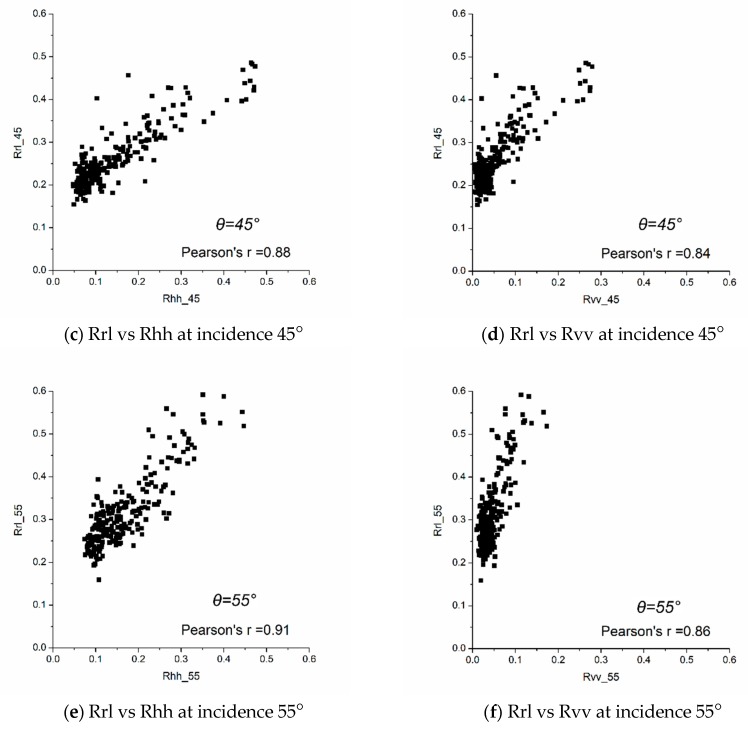
Correlation between GNSS LHCP reflectivity, Rrl, and ELBARA-II horizontal/vertical reflectivity, Rhh/Rvv, for three different incidence angles, namely 35°, 45°, and 55°, respectively, from top to bottom.

By contrasting GNSS-R and radiometer data, a significant correlation was found between the LHCP reflectivity measured by GNSS-R and the horizontal/vertical liner polarization reflectivity measured by the radiometer. Figure 8 displays Oceanpal GNSS reflectivity and ELBARA-II reflectivity measurements for different incidence angles (35°~55°). The Pearson correlation coefficient between GNSS LHCP reflectivity (Rrl) and ELBARA-II horizontal reflectivity (Rhh) is higher than the correlation coefficient between GNSS LHCP reflectivity (Rrl) and ELBARA-II vertical reflectivity (Rvv).

### 3.3. Neural Net Fitting Results

**Figure 9:**
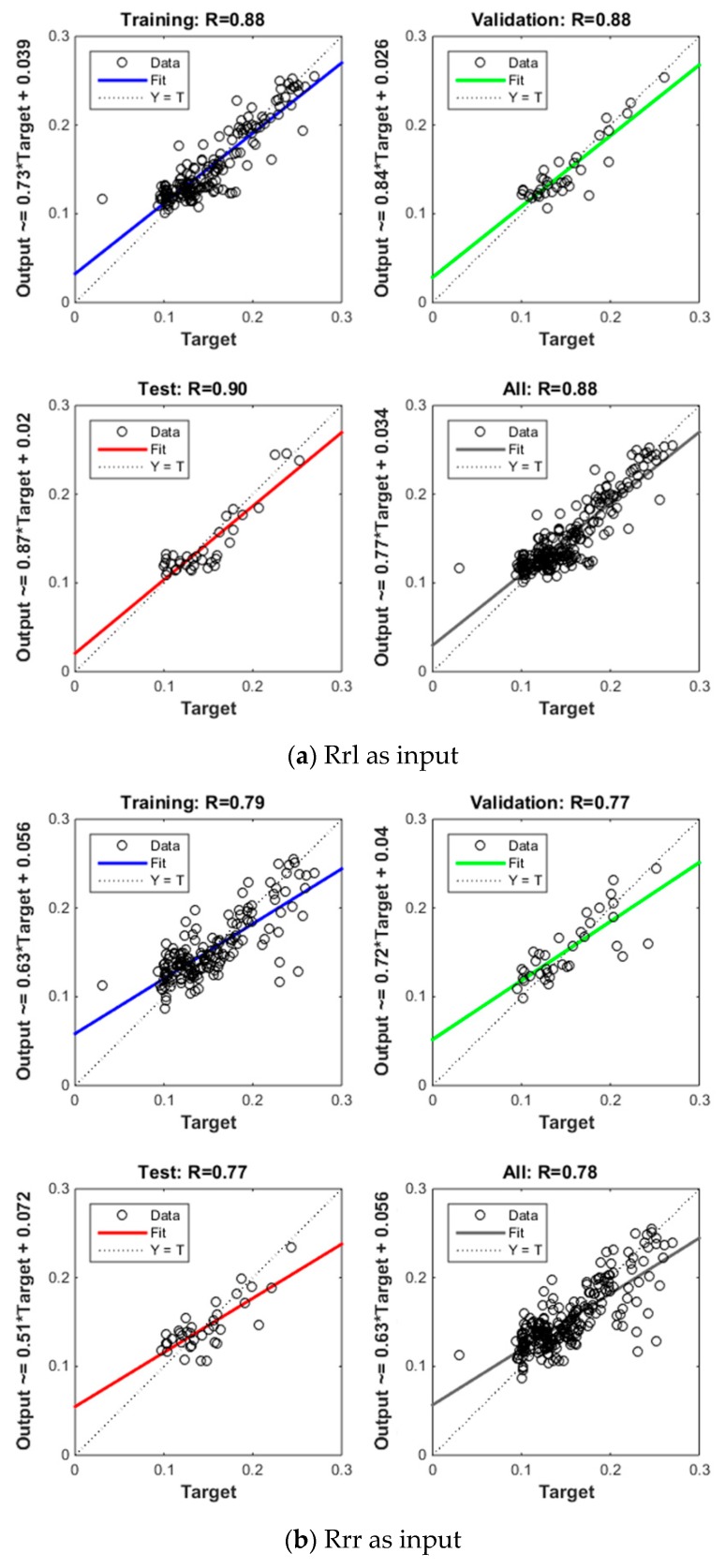

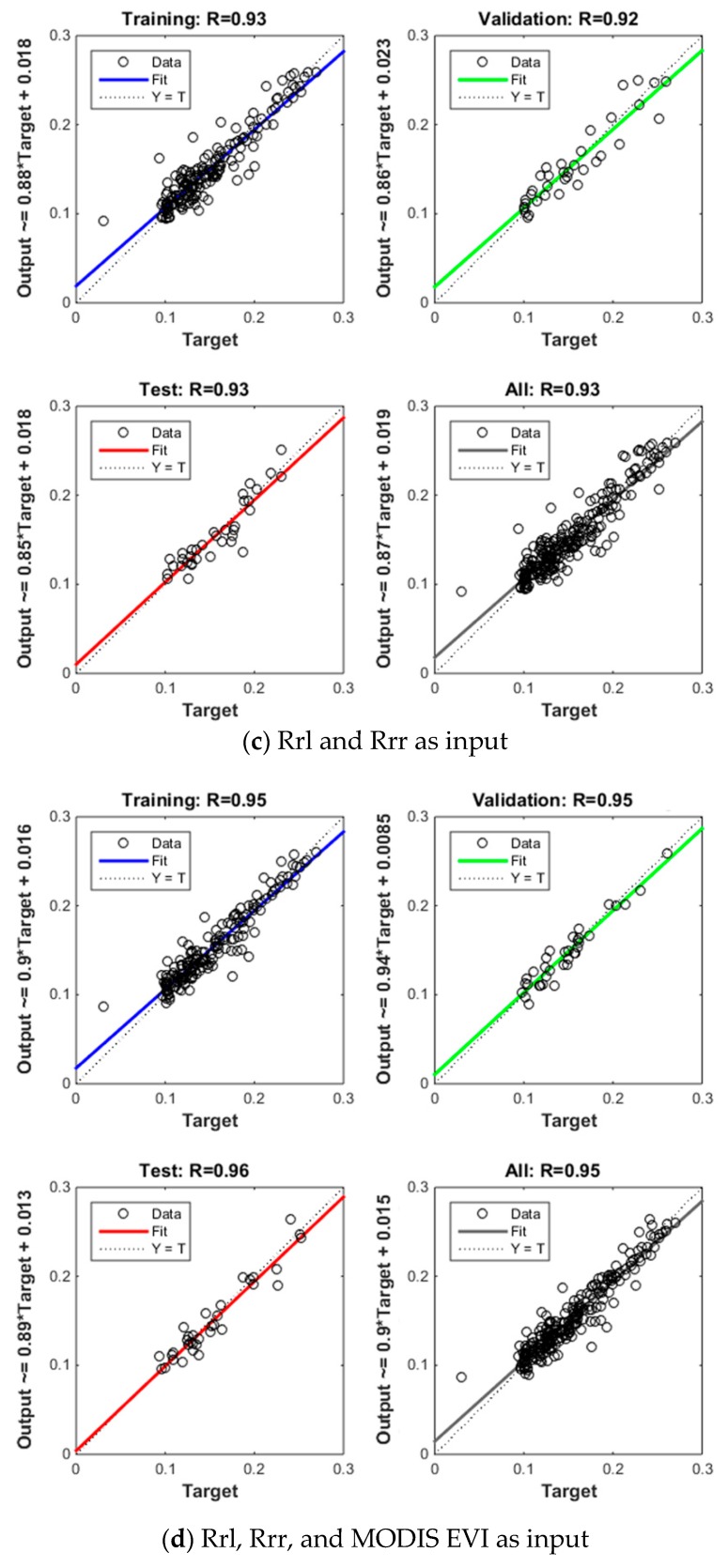
Regression of GNSS-R Neural Net Fitting.

Figure 9 shows the neural net fitting results and Table 2 shows the regression coefficients and RMSEs of the four neural networks. In the first network, the input was GNSS LHCP reflectivity for 10 different incidence angles, from 20° to 65° in 5° intervals. The regression was 0.90 for the test samples, and 0.88 for all the samples. The root mean squared error (RMSE) between GNSS-R measurements and in situ soil moisture was 0.02 m^3^/m^3^ (Figure 9a). Then Rrr was set as input, the regression and RMSE were about 0.78 and 0.027 m^3^/m^3^, respectively, under the same conditions (Figure 9b). When Rrr was added together with Rrl as input, the regression improved to 0.92 and the RMSE was reduced to 0.016 m^3^/m^3^ (Figure 9c). Finally, EVI from MODIS was added as input together with Rrl and Rrr, and the regression improved to 0.94 and the RMSE was reduced to 0.014 m^3^/m^3^ (Figure 9d). 

### 3.4. Intercomparison Between L-Band Soil Moisture Retrievals and In-Situ Measurements

**Figure 10:**
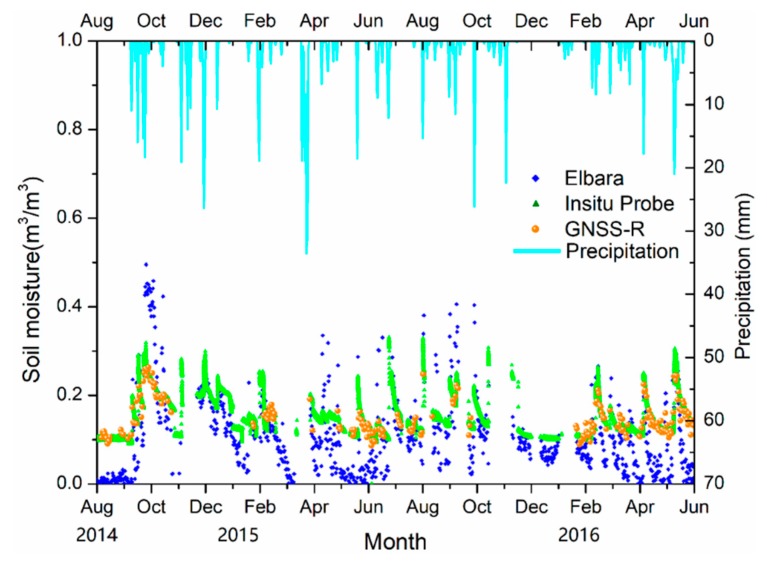
GNSS-R soil moisture as compared to ELBARA-II and ThetaProbe soil moisture as reference.

The soil moisture retrievals during the observation period of August 2014 to May 2016 were compared to soil moisture measured by in situ probes. As illustrated in Figure 10, the soil moisture results retrieved by GNSS-R are in good agreement with the measurements by the soil moisture probe. The correlation coefficient between ELBARA-II and GNSS soil moisture is 0.83, and 0.81 between ELBARA-II and in-situ probes. The statistic errors between the retrieved soil moisture and in situ measurements are listed in Table 3. Soil moisture retrieved by both L-band remote sensing methods have the same variation tendency and are sensitive to the influence of precipitation. 

## 4. Discussion and Conclusions

In this paper, the measurements of soil moisture with two different L-band observation systems are presented. On the one hand, the ESA ELBARA-II passive radiometer measures brightness temperature in horizontal/vertical polarization, from where reflectivity on both linear polarizations may be derived. On the other hand, the ESA Oceanpal GNSS Reflectometry instrument measures soil surface reflectivity on both LHCP/RHCP circular polarizations. 

GNSS surface-measured reflectivity basically depends on soil moisture and incidence angle. Thus, reflectivity under different incidence angles has been used to obtain soil moisture. GPS signals are transmitted with right hand circular polarization (RHCP), but when reflecting from the earth surface, the electromagnetic waves suffer a polarization rotation, and most of the power is reflected in LHCP. Thus, Rrl is more sensitive to soil moisture variation than Rrr. Although Rrr was less sensitive to soil moisture, when Rrr is set together with Rrl as input, the regression to obtain soil moisture improves. Moreover, when EVI from MODIS was added as input together with Rrl and Rrr, the regression was further improved to 0.94 and the RMSE was reduced to 0.014 m^3^/m^3^, which indicates that the vegetation effect is significant in the L-band soil moisture inversion.

This study uses data measured with an Oceanpal GNSS-R system at the Valencia anchor station. Reference soil moisture estimations were obtained from the ELBARA-II radiometer and in situ measurements. There was a strong correlation between the GNSS-R LHCP reflectivity and horizontal/vertical reflectivity calculated from ELBARA-II radiometer measurements. A neural net fitting was used for GNSS-R soil moisture inversion. Daily averaged GNSS LHCP reflectivity was used for 10 different incidence angles as input, and in situ soil moisture measurements as the target. The results clearly show that the algorithm is effective. Going still further, adding Rrr and MODIS EVI as input significantly improved the accuracy of the results.

## Figures and Tables

**Table 1:** Data Acquisition Schedule.

Measurement Mode	Channels Information
1-LD (3 min)	Channel 1: Reflected LHCP
	Channel 2: Direct RHCP
2-DR (0.5 min)	Channel 1: Direct RHCP
	Channel 2: Reflected RHCP

**Table 2:** Regression coefficient and RMSE of 4 neural networks.

Input	Regression Coefficient	RMSE (m^3^/m^3^)
***Rrl***	0.88	0.020
***Rrr***	0.78	0.027
***Rrl* and *Rrr***	0.93	0.016
***Rrl, Rrr,* and EVI**	0.95	0.014

**Table 3:** Statistic errors between the retrieved soil moisture and in situ measurements.

	R^2^	*p*-Value	Bias (m^3^/m^3^)	RMSE (m^3^/m^3^)	UbRMSE (m^3^/m^3^)
Oceanpal	0.9012	<0.0001	0.005	0.0137	0.0128
Elbara	0.6499	<0.0001	0.041	0.0841	0.0734
